# Investigating microglia-neuron crosstalk by characterizing microglial contamination in human and mouse patch-seq datasets

**DOI:** 10.1016/j.isci.2023.107329

**Published:** 2023-07-11

**Authors:** Keon Arbabi, Yiyue Jiang, Derek Howard, Anukrati Nigam, Wataru Inoue, Guillermo Gonzalez-Burgos, Daniel Felsky, Shreejoy J. Tripathy

**Affiliations:** 1 The Krembil Centre for Neuroinformatics, Centre for Addiction and Mental Health, Toronto, ON, Canada; 2 Institute of Medical Science, Temerty Faculty of Medicine, University of Toronto, Toronto, ON, Canada; 3 Department of Immunology, University of Toronto, Toronto, ON, Canada; 4 Robarts Research Institute, Western University, London, Canada; 5 Department of Physiology and Pharmacology, Schulich School of Medicine and Dentistry, Western University, London, Canada; 6 Translational Neuroscience Program, Department of Psychiatry, University of Pittsburgh, 3811 O’Hara Street, Pittsburgh, PA 15213, USA; 7 Department of Psychiatry, University of Toronto, Toronto, ON, Canada; 8 Division of Biostatistics, Dalla Lana School of Public Health, University of Toronto, Toronto, ON, Canada; 9 Department of Physiology, University of Toronto, Toronto, ON, Canada

**Keywords:** Cellular neuroscience, Techniques in neuroscience, Transcriptomics

## Abstract

Microglia are cells with diverse roles, including the regulation of neuronal excitability. We leveraged Patch-seq to assess the presence and effects of microglia in the local microenvironment of recorded neurons. We first quantified the amounts of microglial transcripts in three Patch-seq datasets of human and mouse neocortical neurons, observing extensive contamination. Variation in microglial contamination was explained foremost by donor identity, particularly in human samples, and additionally by neuronal cell type identity in mice. Gene set enrichment analysis suggests that microglial contamination is reflective of activated microglia, and that these transcriptional signatures are distinct from those captured via single-nucleus RNA-seq. Finally, neurons with greater microglial contamination differed markedly in their electrophysiological characteristics, including lowered input resistances and more depolarized action potential thresholds. Our results generalize beyond Patch-seq to suggest that activated microglia may be widely present across brain slice preparations and contribute to neuron- and donor-related electrophysiological variability *in vitro*.

## Highlights

• Microglial transcripts in neuronal Patch-seq data measured as contamination score
• Contamination varied by donor and cell type, was reflective of activated microglia
• Neurons with greater contamination had lower input resistances and AP thresholds
• Microglia contribute to electrophysiological variability *in vitro*

## Graphical abstract

**Figure:**
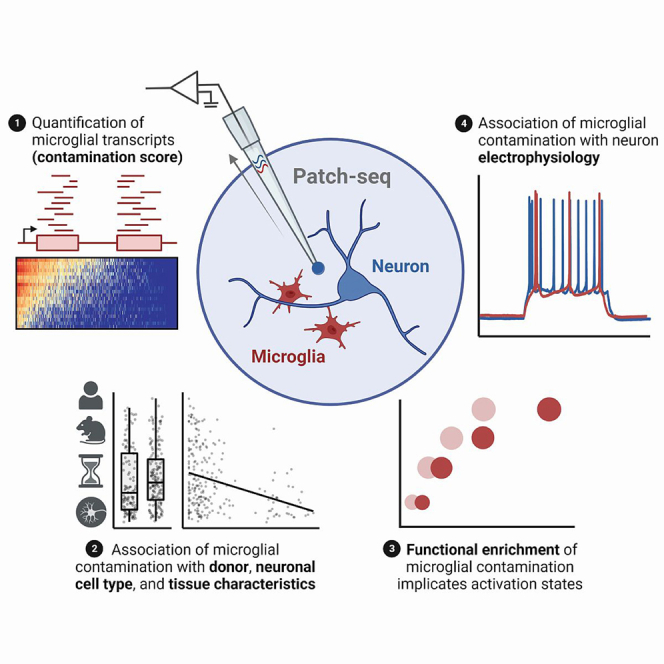

## Introduction

Microglia are the innate immune cells of the central nervous system and exhibit a complex array of phenotypes and functions.^1^^,^^2^ Microglia frequently interact with neurons and have well-established roles in sculpting synaptic connections and regulating their plasticity.^3^ For example, pruning synapses is crucial for the refinement of neuronal circuitry during development as well as learning and memory in the adult brain. Synaptic pruning is regulated by phagocytic microglia and several immune-related signaling pathways,^4^^,^^5^^,^^6^ and has important emerging causal links to neuropsychiatric disease.^7^^,^^8^ The strength of retained functional synapses is modified by microglia through the release of various cytokines.^9^^,^^10^^,^^11^^,^^12^^,^^13^^,^^14^

There is growing evidence that microglia function beyond the synapse to regulate aspects of intrinsic neuronal excitability. Microglia actively survey neural circuit excitability and can undertake a role similar to inhibitory cells in maintaining the synchrony and homeostasis of local neuron activity.^15^ Microglia achieve this by forming direct contacts with neuronal cell bodies^16^^,^^17^ or by secreting specific molecules such as ATP, TNF-α, IL1B, and other cytokines.^18^ However, the effects of this regulation are highly varied among different neuronal types and brain regions. For example, microglial activation decreases intrinsic excitability among neocortical pyramidal cells,^19^ but increases excitability among cerebellar Purkinje cells via phosphatase-dependent signaling and SK channel regulation.^20^ Microglia further differ in their transcriptomic states across neocortical layers, with differences in the densities of homeostatic and proliferative microglial subtypes in upper relative to lower layers.^21^ Despite the importance of microglia for neuronal function, much of this work has been done using rodent models, and it is unclear how such effects might translate to humans or vary across diverse neuronal types.

Patch-seq is a recently developed method for characterizing multi-modal neuronal diversity by combining patch-clamp electrophysiology with single-cell RNA-sequencing (scRNA-seq).^22^ Unlike more traditional methods for scRNA-seq that first rely on cell dissociation, Patch-seq is unique in that the patch pipette is used to carefully harvest mRNA from the cytoplasm and nucleus of the targeted neuron following electrophysiology.^22^^,^^23^ A challenge with Patch-seq is mRNA contamination from surrounding cells in the local microenvironment. We initially reported the paradoxical presence of high levels of gene expression markers of non-neuronal cells,^24^ including microglia, in multiple Patch-seq datasets of confirmed neurons.^25^^,^^26^^,^^27^ Because this contamination was only observed in datasets collected from acute brain slices but not those from sparsely plated cell cultures,^28^ we reasoned that such contamination is likely due to the physical contact of the patch pipette with processes of other cells in the local microenvironment of the recorded neuron. Since our initial report, a number of Patch-seq studies have corroborated these findings, and in particular, that microglial transcripts appear especially prevalent among neuronal Patch-seq datasets.^29^^,^^30^^,^^31^ However, it remains unknown whether contamination from surrounding microglia is associated with functional characteristics of the recorded neurons.

Here, we explored two related goals. First, we sought to comprehensively characterize the phenomenon of microglial contamination in large neuronal Patch-seq datasets from humans and mice. For example, how prevalent and extensive is microglial contamination in these datasets? And what is the transcriptomic signature of such contamination? Second, rather than treating this contamination as a mere nuisance, we leverage it to gain insights into how microglia are co-localized with and might further be interacting with neurons. For example, is microglia contamination in Patch-seq more associated with some neuron types than others? And does it tend to differ between human neurosurgical donors? Critically, we investigate whether microglial contamination is associated with altered neuronal electrophysiological characteristics. Collectively, our findings indicate that microglial contamination in Patch-seq is reflective of activated microglia in the local microenvironment and is further associated with specific alterations in neuronal electrophysiology.

## Results

### Strategy for assessing the impact of microglial contamination in neuronal patch-seq samples

**Figure 1:**
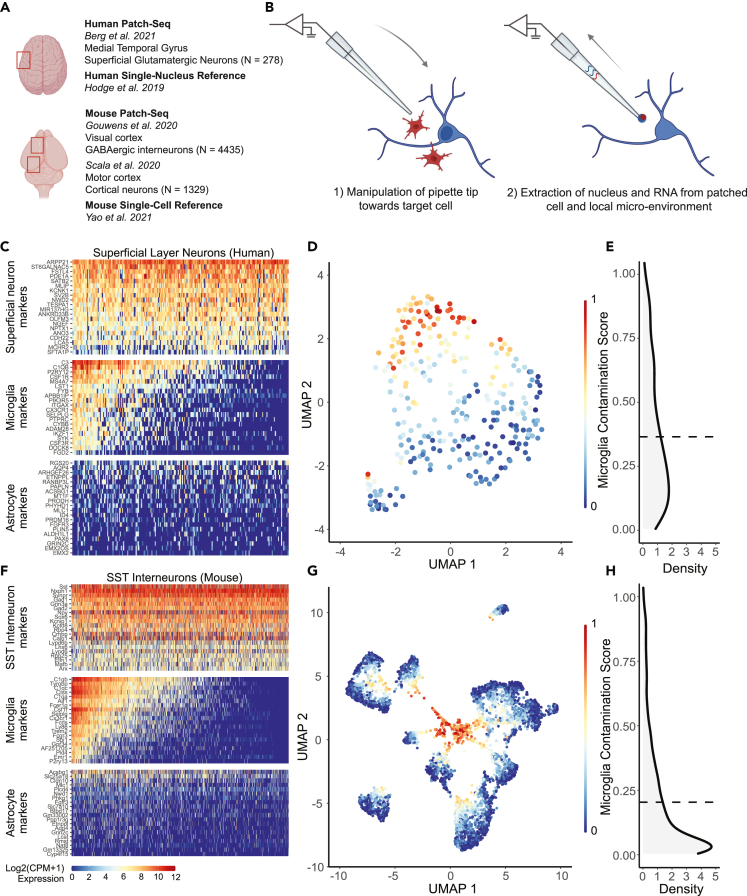
Patch-seq transcriptomes of human and mouse neurons express microglial marker genes at levels sufficient to drive unbiased clustering (A) Overview of datasets used in the current study, including three recent Patch-seq datasets of cortical neurons in human and mouse, with comparisons made to dissociated human single nucleus and mouse single cell RNA-sequencing datasets, respectively. (B) Schematic illustrating manipulation of patch-pipette toward a neuron of interest, and collection of mRNA from cell nucleus, cytoplasm, and possibly the surrounding environment via the patch-pipette. (C and F) Gene expression profiles for human superficial glutamatergic neurons (C, Berg dataset) or mouse SST interneurons (F, Gouwens dataset) for various cell type-specific markers. Each row represents a cell type-specific marker gene and columns represent individual neurons, ordered from left to right by decreasing microglial contamination score. (D and G) Low-dimensional visualization of transcriptomes from neuronal Patch-seq samples clustered by most variable gene expression and color-coded by microglial contamination score for human pyramidal cells (D, Berg dataset), and mouse GABAergic interneurons (G, Gouwens dataset). (E and H) Distribution density of microglial contamination scores for human (E) and mouse (H) neurons, dashed lines indicate population mean. (See also Figure S1 for Scala dataset).

We define microglial contamination as the number of microglial transcripts sampled by Patch-seq relative to the number expected by cell dissociation-based experiments (see STAR Methods). To study the extent and potential impact of microglial contamination in Patch-seq experiments, we used three datasets published previously (Figure 1A and Table 1), including (1) human supragranular glutamatergic neurons from the medial temporal gyrus,^29^ (2) mouse GABAergic interneurons from the primary visual cortex,^32^ and (3) mouse glutamatergic and GABAergic neurons from the primary motor cortex.^31^ These datasets were selected in part due to their large size, reflecting hundreds to thousands of neuronal samples. In addition, these datasets are considered to be of high quality, reflecting thousands of measured genes per neuronal sample, as they follow extensive internal optimization procedures and strict transcriptomic and electrophysiological data quality control.^30^^,^^33^ Lastly, the availability of high-quality cell dissociation–based single-cell and single-nucleus transcriptomes from parallel samples (Figure 1A; Hodge et al., 2019; Yao et al., 2021) enables rigorous comparisons between Patch-seq based and cell dissociation-based gene expression profiles.

**Table 1:** Description of Patch-seq datasets used in this study

Dataset Reference	Species	Brain Region	Cell types sampled	N cells included	N donors/animals included
Berg et al.^29^	Human	Medial Temporal Gyrus	Superficial pyramidal cells	278	48
Gouwens et al.^32^	Mouse	Visual cortex	GABAergic cells	4,270	985
Scala et al.^31^	Mouse	Motor cortex	Glutamatergic and GABAergic cells	1,329	266

### Microglial contamination is widely present in human and mouse patch-seq neuronal transcriptomes and drives unbiased clustering

To quantify the levels of microglial contamination in Patch-seq-sampled neurons, we first examined the expression of cell type-specific marker genes. Patch-seq neuronal samples expressed high levels of the expected marker genes; for example, *SV2B* and *SATB2* in human superficial pyramidal cells (Figure 1C, top row) and *Sst*, *GAD1*, and *GAD2* in mouse somatostatin interneurons (Figure 1F, top row). However, many Patch-seq neuronal transcriptomes also express high levels of multiple microglia-specific markers that are not expected to be expressed in neurons.^30^ Such markers include *C3* and *C1QB* in human samples (Figure 1C, middle row) and, additionally, *Tyrobp* in mouse samples (Figure 1F, middle row). Given the widespread detection of unexpected microglia-specific transcripts in Patch-seq neuronal samples, we reason that this reflects inadvertent sampling of mRNA from microglial cellular processes via the patch-pipette (Figure 1B), as opposed to the endogenous upregulation of microglia-specific transcripts by the sampled neuron. We note that the presence of astrocyte-specific marker transcripts was generally rare in both human and mouse samples (Figures 1C and 1F, bottom row), suggesting that astrocytes are likely not a major source of cellular contamination in these samples. We also found qualitatively similar findings indicating the presence of multiple microglial transcripts among mouse motor cortex glutamatergic and GABAergic Patch-seq neuronal samples among the Scala dataset (Figures S1A and S1B), despite key biological and technical differences in how these data were collected, for example, electrophysiological recordings being performed at room temperature in the absence of blockers of synaptic transmission.^31^

To obtain a single value quantifying the extent of microglial contamination, we calculated microglial contamination scores for each Patch-seq neuronal cell, as defined previously by our group and others.^24^^,^^30^ These scores provide an interpretable scalar value between 0 and 1, where 0 indicates little to no detected microglial contamination and 1 indicates a high degree of contamination at levels similar to those expressed by microglia sampled via single-nucleus (human) or single-cell (mouse) RNA-seq. While our definition of these microglial contamination scores is in part dependent on transcriptomic cell type identity for the purpose of normalization (see STAR Methods), in practice, we note that these normalized contamination scores are strongly correlated with the overall level of marker gene expression of microglial transcripts in each cell (Figure S4A).

We asked if microglial contamination is sufficient to drive unbiased transcriptional clustering of Patch-seq neuronal samples. Following standard workflows for single-cell analyses (see STAR Methods), we found that human and mouse Patch-seq neuronal samples exhibited strong visual evidence of transcriptomic clustering, in part, according to their levels of microglial contamination (Figures 1D, 1G, and S1C). On average, we found that human Patch-seq neuronal transcriptomes appeared considerably more contaminated by microglia than in the mouse datasets (Figures 1E, 1H, and S1D; human: 0.34 ± 0.25, mouse, Gouwens: 0.19 ± 0.22; mouse, Scala, glutamatergic: 0.15 ± 0.14; Scala, GABAergic: 0.14 ± 0.14, mean ± SD, normalized microglial contamination scores).

### Inter-donor differences and neuronal identity explain the most variation in microglial contamination

**Figure 2:**
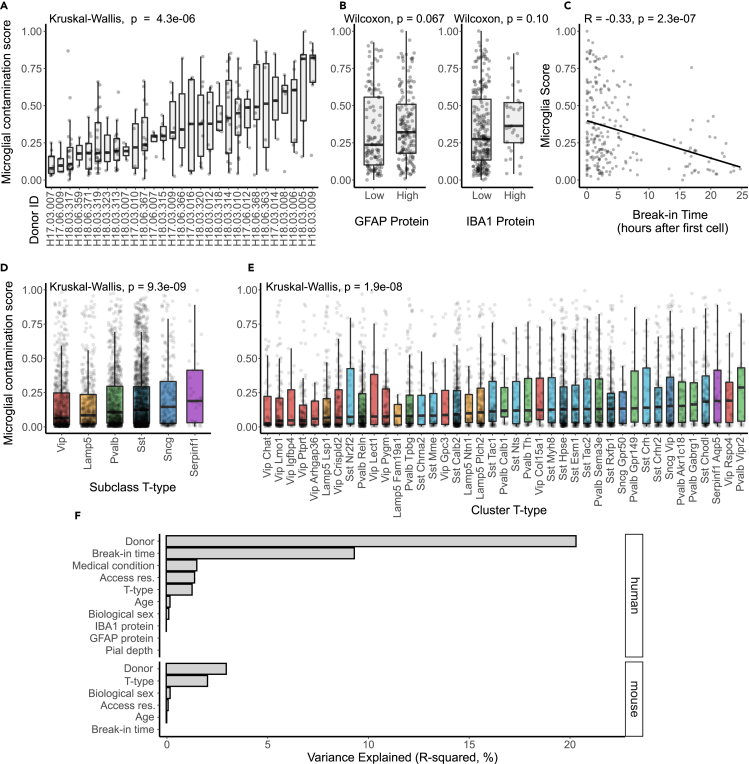
Associations between donor, cell type, and tissue characteristics with microglial expression in Patch-seq samples (A–C) Associations between microglial contamination scores (y axis) estimated from human pyramidal neuron Patch-seq samples and neurosurgical donor identity (A), GFAP (left) and IBA1 (right) protein expression assayed via immunohistochemistry (low ≤1, high >1) (B), and the time at which the cell was patched relative to the first donor’s cell (break-in time, hours) (C). p values are reported (Wilcoxon, Kruskal-Wallis). (D and E) Associations between microglial contamination scores (y axis) estimated from mouse GABAergic interneuron Patch-seq samples and interneuron cell type identity, summarized at either the subclass level (D) or cluster level (E). (F) Estimated percent variance explained (R-squared) in microglial contamination scores among human (top) and mouse Gouwens dataset samples (bottom) by various factors modeled together, including donor/animal identity, neuronal cell type identity (t-type), age, sex, break-in time, access resistance, sample depth from pial surface (pial depth), medical condition (epilepsy or tumor), IBA1 or GFAP protein expression. (See also Figure S5 for Scala dataset).

We next wanted to understand how experimental characteristics, like cell type identity, donor characteristics, and tissue quality, might correlate with microglial contamination in Patch-seq cells. Among human Patch-seq sampled superficial pyramidal neurons, we found samples collected from different neurosurgical donors varied considerably in the levels of microglial contamination (Figure 2A, Kruskal-Wallis ANOVA p = 4.3 ∗ 10^−6^). Utilizing data from immunohistology performed on brain slices not used for electrophysiological characterization, IBA1 and GFAP protein expression (markers of microglia and astrocytes, respectively) was associated with increased microglial contamination (GFAP: Figure 2B left, Wilcoxon p = 0.067; IBA1: Figure 2B right, Wilcoxon p = 0.10). While the prior association between transcriptomically inferred microglial contamination and IBA1 protein expression is marginal, this serves as an independent confirmation of our interpretation of increased microglial expression in samples from these donors. Given that human neocortical brain slices are often viable for many hours following surgical resection, we asked how differences in times when neurons were recorded from each donor (termed “break-in time”) are associated with microglial contamination. We found a strong negative association between a neuron’s relative time of recording and microglial contamination, with later recorded cells showing less microglial contamination than earlier recorded cells (Figure 2C; R = −0.33, p = 2.3 x 10^−7^; Figure S2A shows same results stratified by individual donors). Finally, we found a trend for cells with higher electrophysiological seal resistances (i.e., a measure of improved recording quality) to show lower amounts of microglial contamination (R = −0.15, p = 0.014, Figure S2B).

Among Patch-seq sampled GABAergic cortical interneurons from mice from the Gouwens dataset, we found that different cell types exhibited different levels of microglial contamination (subclass: Figure 2D, cluster: Figure 2E). We observed lower levels of microglial contamination among Vip and Lamp5 subclasses and comparatively higher levels in Sst, Sncg, and Serpinf subclasses (Figure 2D, Kruskal-Wallis ANOVA p = 9.3 ∗ 10^−9^). Even among more fine-grained neuronal subtypes (i.e., cluster t-type), we observed considerably different levels of microglial contamination among clusters from the same parent subclass (Figure 2E, Kruskal-Wallis ANOVA p = 1.9 ∗ 10^−8^). We further observed differences in microglial contamination stratified by the neocortical layer (Figure S3), although some of these differences are explained by the cell type associations described above. We note that these results and their interpretation have limitations. For mice, we note that animal identity is confounded by transcriptomic type given the use of different mouse transgenic lines to target different cell types. To mitigate the concern that our calculation of the microglial contamination score is in part dependent on transcriptomic cell type identity (see STAR Methods), we performed an additional analysis where we assessed the total levels of expression of microglial markers in each cell and cell type, which revealed similar differences in levels of microglial gene expression across GABAergic subclasses and t-types (Figures S4B and S4C).

For Patch-seq sampled GABAergic and glutamatergic neurons from the Scala mouse dataset, we observed that transcriptomic cell type identity was also associated microglial contamination (Figure S5C, GABAergic: Kruskal-Wallis ANOVA p = 0.097, glutamatergic: Kruskal-Wallis ANOVA p = 1.8 ∗ 10^−12^). Moreover, we observed a modest correlation between levels of microglial contamination among matched GABAergic cell types in both the Gouwens and Scala datasets (Figure S5D, Pearson’s R = 0.38, p = 0.055). Intriguingly, both datasets showed the highest levels of microglial contamination among Pvalb Vipr2 cells (i.e., chandelier cells,^32^ suggesting that chandelier cells might show some inherent differential vulnerability to contact by surrounding microglia (see Discussion). In addition, we found that neurons recorded deeper from the pial surface displayed less microglia contamination (Figure S5A, GABAergic: Pearson’s R = −0.15, p = 0.00012; glutamatergic: Pearson’s R = −0.27, p = 5.4 ∗ 10^−8^), consistent with evidence suggesting greater densities of microglia in upper relative to lower cortical layers.^21^

To further quantify associations between these experimental variables and microglial contamination, we used a random effects model allowing us to assess correlations between Patch-seq sampled neurons recorded from the same donor or animal (see STAR Methods). Among the human pyramidal neuron samples (Figure 2F, top), donor identity explained the most neuron-to-neuron variability in microglial contamination (R^2^ = 20.3%), followed next by break-in time (R^2^ = 9.25%), transcriptomic cellular identity (R^2^ = 2.40%), neuron seal resistance (R^2^ = 2.16), and donor medical condition (R^2^ = 1.66). Other factors, such as donor age at time of surgery, sex, and cell soma depth from pia, were only weakly explanatory for microglial contamination (R^2^ < 1%).

Among mouse samples in the Gouwens dataset (Figure 2F, bottom), we found that the most important factor associated with microglial contamination was donor/animal identity (R^2^ = 2.98%) followed by cell type identity (R^2^ = 2.05) and animal sex, age, break-in time and access resistance explained comparatively less variability (R^2^ < 1%). Among mouse samples in the Scala dataset, we found results qualitatively similar to those in the human dataset (Figure S5E), with animal identity explaining the most variance, particularly in glutamatergic cells (GABAergic R^2^ = 4.99%; glutamatergic R^2^ = 15.1%), followed next by cell type identity (GABAergic R^2^ = 3.55%; glutamatergic R^2^ = 4.45%), with other factors, such as age, sex, cell soma pial depth explaining <2% of variance, each. In total, considerably more neuron-to-neuron variation in microglial contamination could be explained for human samples relative to each of the mouse datasets (R^2^ = 35.6% for human, R^2^ = 5.27% for mice in the Gouwens dataset), perhaps in part reflecting the overall degree of greater microglial contamination among the human samples.

### The microglial transcriptomic signature in Patch-seq is reflective of a distinct neuroinflammatory state

We next sought to identify any distinctive transcriptomic characteristics of microglial contamination in Patch-seq. In particular, we asked whether transcripts concomitantly sampled with neurons reflect particular microglia cell states, such as those observed in disease or in response to injury, or those known to alter neuronal function. For these analyses, we focus on Patch-seq datasets (Berg and Gouwens) collected by the Allen Institute for Brain Science, allowing direct comparison of these transcriptomes to those from dissociated nuclei and cells collected at the same facility using similar methods (see STAR Methods).

**Figure 3:**
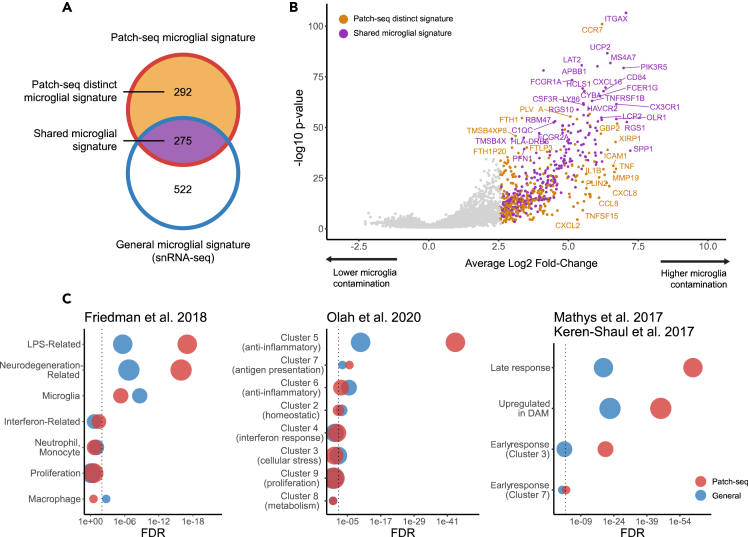
Microglial contamination in human Patch-seq reflects a distinct transcriptional signature related to microglia activation (A) Venn diagram indicating the number of genes defining various transcriptional signatures: the signature of general microglia in human dissociated single-nucleus data (blue border), the signature of Patch-seq microglial contamination in human neuronal Patch-seq data (red border), genes that are shared between the Patch-seq microglial and general microglia signatures (purple fill), and genes that are distinct to the Patch-seq microglial signature that are not also present in the transcriptional signatures of general microglia (yellow fill). (B) Volcano plot of transcriptional signature of Patch-seq microglial contamination, illustrating differentially expressed genes in Patch-seq datasets between human neuronal samples with high vs. low microglial contamination. Points denote differentially expressed genes (log2 fold-change >2.5; p value <0.01), and colors are as in (A). (C) Enrichment analysis of general microglia (blue) and Patch-seq microglia (red) transcriptional signatures (as in A) intersected with gene sets of diverse microglial phenotypes and states from multiple data sources (titles indicated). Dot size reflects the number of genes in each gene set. The dotted line is FDR = 0.05. (See also Figure S6 for Gouwens mouse data).

To determine a *“transcriptomic signature of Patch-seq microglial contamination”*, we contrasted transcriptomes from neuronal samples with the highest microglial contamination to those with the lowest contamination. Numerous genes had significantly increased transcript amounts in Patch-seq neurons with high contamination (log2 fold-change >2.5; p value <0.01), with 567 genes in human samples (Figure 3A and Table S1) and 422 genes in mouse samples (Figure S6 and Table S2). Crucially, this analysis revealed a number of genes with high expression levels in Patch-seq samples that are further known to mediate how microglia regulate aspects of neuronal excitability, including TNF, IL1B, IL6, and CCL2.^18^ These example genes are particularly salient as they were not also used in defining microglial contamination scores.

Next, we defined a *“general microglia transcriptional signature”*, where we used available gene expression profiles from dissociated single-nucleus (human) or single-cell (mouse) RNA-seq to identify genes overexpressed in microglia compared to neurons. We identified 797 such genes in human and 1,276 genes overexpressed in mouse microglia. Lastly, we defined a “*Patch-seq distinct microglial signature”* by identifying genes from the *“transcriptomic signature of Patch-seq microglial contamination”* signature that did not overlap with the *“general microglia transcriptional signature”*. Many genes were identified in the Patch-seq distinct microglial signature that appeared specific to samples with high levels of microglial contamination in Patch-seq, but not dissociated microglia relative to neurons. Specifically, we saw 292 such genes in humans (Figures 3A) and 46 such genes in mice (Figure S6A). While this analysis may suggest distinct transcriptional signatures between humans and mice, there is likely a greater difference between microglial transcripts sampled via Patch-seq compared to those sampled via single-nucleus (human) versus single-cell dissociation (mouse). Recent reports have indicated that single-nucleus RNA-seq is especially limited in the context of microglia-related analyses.^34^

To elaborate on the transcriptional signatures associated with Patch-seq microglial contamination, we performed enrichment analyses of several published reference gene sets that capture diverse microglial states. We compared Patch-seq signatures to our general microglial signatures, to better understand how they may differ. Among human Patch-seq samples, we found evidence for an activated, inflammation-related microglial signature among Patch-seq samples with high microglial contamination. Specifically, the transcriptomic signature of Patch-seq microglial contamination was considerably more enriched for LPS-related (FDR = 1.0 ∗ 10^−17^) and neurodegeneration-related (FDR = 1.2 ∗ 10^−16^) gene signatures defined in Friedman et al.^35^ compared to the general microglia transcriptional signature (Figure 3C). Relative to microglia clusters identified in scRNA-seq from aged and Alzheimer’s human samples,^36^ the Patch-seq microglial signature was highly enriched for microglia-specific clusters 5, reflecting anti-inflammatory responses (FDR = 8.7 ∗ 10^−45^). There was also enrichment of microglial late response genes (FDR = 1.2 ∗ 10^−60^) and disease-associated microglia (DAM, FDR = 4.9 ∗ 10^−46^) described previously.^37^^,^^38^ In mouse, we observed few major differences in enrichments between the Patch-seq microglial signature and the general microglial signature (Figure S6C), again suggesting fewer differences between microglia sampled during Patch-seq and reference microglial transcriptomes. In summary, these analyses point to an activated, inflammation-related microglial signature among Patch-seq samples with high microglial contamination, which in part appears distinct from microglial signatures sampled via single-nucleus RNA-seq.

### Microglial contamination is associated with alterations in intrinsic electrophysiology

Lastly, we next wanted to assess whether microglial contamination in Patch-seq is associated with altered excitability of the recorded neuron. Because each Patch-seq sampled neuron was first characterized for its intrinsic electrophysiological characteristics prior to mRNA harvesting, we were able to leverage these data to ask how microglial contamination might be associated with cell-to-cell variability in electrophysiological features across neuron subtypes and species.

**Figure 4:**
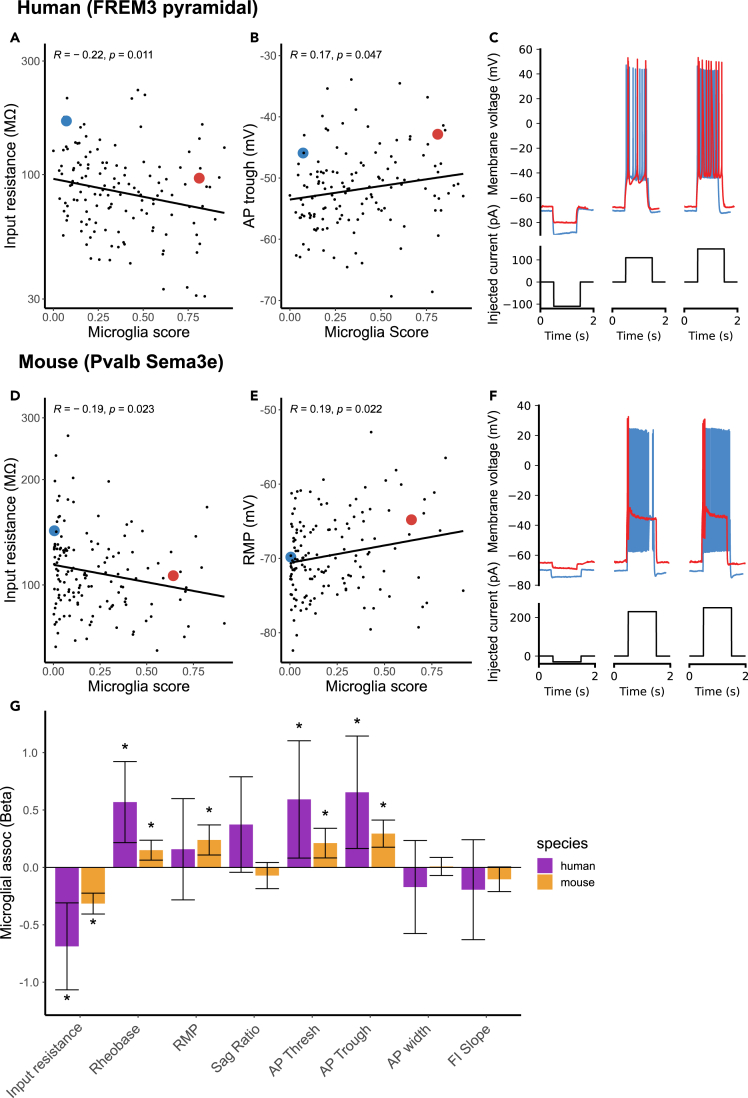
Microglial contamination is associated with altered neuronal intrinsic electrophysiology (A and B) Scatterplots illustrating cellular input resistances (y axis, A) and action potential trough (y axis, B) versus microglial contamination scores (x axis). Each dot reflects one human FREM3 pyramidal cell sampled via Patch-seq. Inset correlations reflect Pearson’s correlations and line indicates best linear fit. Inset blue and red dots reflect two pyramidal cells matched by neurosurgical donor, cell type (FREM3), and cortical depths, but with differing levels of transcriptomically inferred microglial contamination. (C) Membrane voltage traces (top) and corresponding injected currents (bottom) for neurons highlighted in A, reflecting different subthreshold (left) and suprathreshold (middle, right) characteristics of exemplar cells. (D–F) Same as (A-C) for mouse Pvalb Sema3e cells from the Gouwens dataset. Resting membrane potentials are shown on y axis in (E). (G) Association between microglial contamination and electrophysiological characteristics, as estimated using a mixed effects model. Bars indicate effect sizes (Beta coefficients) and error bars denote 95% confidence intervals. Asterisks denote beta coefficients where p < 0.05 (ANOVA). Positive (negative) beta coefficients indicate increased microglial contamination is associated with an increase (decrease) in the electrophysiological property. Electrophysiological features have been standardized to unit variance, enabling comparison of beta coefficient effect sizes between species. (See also Figure S7 for Scala dataset).

For the purposes of illustration, we first consider a representative example of two human FREM3 pyramidal cells (Figure 4C), one with low (0.06 microglial contamination score, Figure 4C, blue traces) and one with high microglial contamination (1.00 microglial contamination score, Figure 4C, red traces), but otherwise matched in their overall characteristics (same neurosurgical donor, neuron cell type, similar cortical depths [355 vs. 331 μm], etc). We observed a number of electrophysiological characteristics that differed between these two representative cells. For example, the more contaminated cell had a lower input resistance (96 vs. 167 MOhms), greater rheobase (110 vs. 70 pA), a more depolarized resting membrane potential (−67.8 vs. −70.6 mV), and more depolarized minimum voltage following an action potential (i.e., AP trough voltage, −42.8 vs. -45.9 mV) than the less contaminated cell, among other differences. We note that these differences are within the expected ranges of variability for these cells^39^^,^^40^ and that the original electrophysiological datasets have undergone strict quality control.^29^ Across human FREM3 pyramidal cells, we also saw that higher microglial contamination scores were associated with lower input resistances (Figure 4A, Pearson’s R = −0.22, p = 0.011) and more depolarized action potential through voltages (Figure 4B, left, Pearson’s R = 0.17, p = 0.047). To provide context for these associations, microglial contamination explained 5.2% of the cell-to-cell variation in input resistance values among human FREM3 pyramidal cells. However, this value is similar to the variance in input resistance explained by the depth of the recorded neuron from the pial surface, 5.1%, noted previously to be a major biological factor distinguishing human superficial pyramidal cells from one another.^29^^,^^39^^,^^40^

As an additional illustrative example, we further considered examples of mouse Pvalb Sema3e cells (reflecting deep layer fast-spiking cells^32^), chosen from the same animal but with one example cell expressing low (0.005 microglial contamination score, Figure 4F, blue traces) and the other higher microglial contamination (0.64 microglial contamination score, Figure 4F, red traces). Among these examples and other cells from the same t-type, we observed that cells with higher microglia contamination displayed decreased input resistances (Figure 4D, Pearson’s R = −0.19, p = 0.023) and more depolarized resting membrane potentials (Figure 4E, Pearson’s R = 0.19, p = 0.022), and greater failure in sustaining high frequency firing during sustained depolarizing current injection (Figure 4F)

To quantify the association between microglial contamination and electrophysiological features more systematically, we used a statistical approach to ask how microglial contamination is associated with cell-to-cell variability in electrophysiological features, using mixed effects models to control for cellular and donor/animal identity (see STAR Methods). Among human pyramidal neurons, we found increased microglial contamination associated with multiple electrophysiological features (Figure 4G, statistics in Table S3). Most strikingly, we found that increased microglial contamination was associated with significantly decreased input resistances (Beta = −0.68, SE = ±0.19, p = 4.5 ∗ 10^−4^). In addition, a number of suprathreshold features, including action potential threshold and trough voltages, were also significantly associated with microglial contamination (Beta = 0.59, SE = ±0.26, p = 0.024; Beta = 0.65, SE = ±0.24, p = 0.0093). Among GABAergic mouse neurons from the Gouwens dataset (Figures 4G and Table S3), we also observed decreased input resistances (Beta = −0.31, SE = ±0.04, p = 1.2 ∗ 10^−11^) and increased action potential threshold and trough voltages (Beta = 0.21, SE = ±0.06, p = 0.0013; Beta = 0.29, SE = ±0.06, p = 1.12 ∗ 10^−6^). We further saw increased microglial contamination associated with more depolarized resting membrane potentials (Beta = 0.24, SE = ±0.07, p = 3.5 ∗ 10^−4^) and a trend toward decreased frequency-current (FI) curve slopes (Beta = −0.10, SE = ±0.05, p = 0.059). We saw similar directions of association between microglia contamination and electrophysiological properties of glutamatergic and GABAergic mouse neurons from the Scala dataset (Figure S7 and Table S4), including decreased input resistances (glutamatergic: Beta = −0.61, SE = ±0.29, p = 0.033, GABAergic: Beta = −0.41, SE = ±0.20, p = 0.049) and increased sag ratios among glutamatergic cells (Beta = 1.0, SE = ±0.31, p = 0.0015). Together, these findings indicate that microglial contamination is associated with specific alterations in neuronal intrinsic electrophysiological characteristics that are replicable across species and laboratories.

## Discussion

Our analyses of three large-sample human and mouse Patch-seq datasets collected from acute brain slices suggest that microglial contamination is widely present in the transcriptomes of the sampled neurons. A number of technical and biological factors were associated with microglial contamination, including donor-specific factors, particularly in human neurosurgical biopsies, and neuronal cell type identity. The transcriptomic signature of microglial contamination in Patch-seq appears indicative of a distinct activated and inflammation-related cell state. Critically, microglial contamination is further associated with altered neuronal electrophysiological features, including lowered input resistances and increased action potential thresholds. Lastly, these results likely generalize beyond Patch-seq to suggest that the effects of microglial contamination may be present to some degree across many acute brain slicing experiments, including those employing patch-clamp electrophysiology.

While the presence of cellular contamination in neuronal Patch-seq datasets has been reported previously, we were somewhat surprised by how prevalent it appeared in the datasets analyzed here. Moreover, we were concerned that microglial contamination appeared to be a major contributor to unbiased clustering of Patch-seq derived neuronal transcriptomes. Microglial contamination differed considerably in samples collected from different donors; in human datasets specifically, this might reflect biological differences, like those related to donors’ need for surgery, including epilepsy or tumor resection. However, such differences may also reflect technical differences, including those related to the inherent challenges in obtaining biopsies from neurosurgical tissue. As such, we note that animal identity was the greatest factor related to microglial contamination among each of the mouse Patch-seq datasets analyzed, suggesting that animal identity may be an important proxy for tissue health or slice quality that may inevitably differ across animals or preparations. Similarly, our analyses of different levels of observed microglial contamination among mouse neocortical neurons might be reflective of differential associations of various cell types to surrounding microglia. For example, we found that Pvalb Vipr2 cells, reflective of chandelier cells, displayed the highest levels of microglial contamination among all tested mouse GABAergic neuronal types across two datasets sampling different neocortical brain regions; intriguingly a recent report suggests that microglia are key regulators of chandelier cell axonal arborization and synapse formation.^41^

We further identified a number of technical factors associated with microglial contamination. For example, we found that in the context of recordings from human neurosurgical biopsies, neurons that were recorded at later times following slice preparation showed considerably lower levels of microglial contamination. However, we note that the interpretation from this analysis is challenging, as it might reflect a lowering of the activation state of local microglia over time but also might be due to other factors related to how cells are sampled (only healthy cells are viable enough to be recorded at later times), so we are careful not to over-interpret this finding. In addition, we noticed that microglial contamination values were qualitatively lower in the Scala dataset relative to the Gouwens dataset. Such differences may reflect biological differences between motor cortex versus visual cortex but may also be due to technical differences, such as recordings from the Scala dataset being performed at room temperature whereas the Gouwens dataset was performed at more physiological temperatures. Lastly, we identified that microglial contamination was associated with factors related to electrophysiological recording quality, including electrode seal resistance. This finding can, in part, explain some of our observed associations between microglial contamination and correlations with intrinsic electrophysiological characteristics. For example, cells with lower seal resistances would be expected to have larger of membrane leak currents, which could contribute to the lower membrane input resistances, higher resting potentials, and greater rheobase currents that we observed associated with cells with greater degrees of microglial contamination.

The transcriptional signature associated with the involvement of microglia in Patch-seq appeared indicative of activated microglia. This inference is based on the detected expression of key hallmark genes, including TNF and CCL2, and that the Patch-seq related microglial transcriptional signature shares similarities with other microglia signatures related to LPS-, neurodegeneration, and disease-associated microglia.^36^^,^^37^^,^^38^ In addition, we note that we saw considerably more microglia-related genes distinct to human versus mouse Patch-seq datasets. While this might be reflective of bona fide species differences, one simple explanation for this finding may be related to our usage of single-nucleus RNA-seq to provide reference profiles of microglia from humans but scRNA-seq from mice. Recently, it has been shown that single-nucleus RNA-seq, when used to profile human microglia, are more prone to missing important transcripts related to microglial proliferation and other disease related processes.^34^ Such transcripts are possibly expressed in distal cellular processes and are likely to be especially present in microglia inadvertently sampled during Patch-seq.

A key question our findings raise is how contact between microglia with neurons, either directly or indirectly, might contribute to altered cellular electrophysiology. While a definitive answer to this question is beyond the scope of this study, we hypothesize two possible explanations that consider the order of events in Patch-seq, where cellular electrophysiology is characterized before mRNA is harvested and sequenced.^22^ First, we hypothesize that microglia might be directly interacting with the characterized neuron, for example, via physical interactions^17^ or through signaling molecules like TNF that induce downstream changes in cellular excitability through phosphatase signaling and calcium-activated potassium channels.^19^^,^^20^ A second alternative hypothesis is the chemotaxis of microglial processes to neurons that have been otherwise altered or damaged, for example by the slice preparation procedure, but such neuron-microglia interactions might not be directly causing alterations to cellular electrophysiology.

Our study raises a number of questions on the nature of microglia-neuron interactions. First and foremost, we need to better understand microglial activation in the context of experimental work making use of acute brain slices. What contributes to increased microglial activation among slices from some donors and animals, but not others? And can unwanted microglial activation be minimized, for example by incorporating known strategies for inhibiting or depleting microglia, like pre-incubating slices with the CSF1R inhibitor, PLX3397^42^^,^^43^? Second, what is drawing microglia to the surrounding microenvironment of neurons undergoing patch-clamp electrophysiology? Are microglial processes chemotaxing to the neuron’s soma (as opposed to synapses or dendrites)? Is the presence of ATP or other known chemoattractants in or near the patch pipette attracting microglia^44^^,^^45^? Are unhealthy neurons damaged by slice preparation or hypoxia releasing pro-inflammatory cytokines that attract microglia? Lastly, and most importantly, to what extent are the microglia-neuronal associations reported here, in the context of acute brain slices, reflective of such interactions *in vivo*? These questions highlight an important potential role for microglia in shaping neuronal excitability and reflect a number of questions for future investigation.

In summary, our results demonstrate that microglia contamination is widely present among neuronal Patch-seq datasets, and furthermore, these observations likely generalize to some degree to many other acute brain slice experiments. However, this contamination presents a novel, unrecognized opportunity for Patch-seq, as our findings suggest that Patch-seq also samples the local cellular microenvironment in immediate proximity to the patch-clamp characterized neuron. Our most provocative results suggest that local microglia are inadvertently activated in a subset of acute brain slice experiments, and moreover, this in turn is associated with alterations in intrinsic electrophysiology of neurons in immediate proximity to such microglia. However, future studies are needed to further understand what biological and technical factors contribute to microglial activation and how such activation could directly contribute to the alterations in neuronal electrophysiology reported here.

### Limitations of the study

Our study has a number of limitations. First, we note that our analyses rely on our ability to reliably infer the presence of microglial processes in proximity to the characterized neuron using transcriptomics.^24^ We feel reasonably confident in this inference as it is unlikely for neuronal cells to endogenously express tens of microglial specific markers and there is a reasonable concordance between microglial mRNA expression and microglia marker protein expression assayed via immunohistochemistry. Second, we note that our analyses are observational in nature, relying on the natural variability inherent to different experiments. As such, it is difficult to conclude cause from effect in our study, for example, whether microglia-neuronal interactions directly contribute to altered intrinsic electrophysiology. Lastly, we were limited in our analyses to the data fields provided by the original study authors. In some cases, this precluded the study of additional, potentially causal factors related to microglial contamination, such as those related to the quality of neurosurgical resection in human subjects.

## STAR★Methods

### Key resources table

**Table:** 

REAGENT or RESOURCE	SOURCE	IDENTIFIER
**Deposited data**		

Patch-Seq of glutamatergic neuron types in layer 2 and layer 3 of the human cortex	Berg et al.,^29^	Allen Institute for Brain Science Cell Types Database
Patch-Seq of neurons in mouse primary visual cortex	Gouwens et al.,^32^	Allen Institute for Brain Science Cell Types Database
Patch-Seq of neurons in mouse motor cortex	Scala et al.,^31^	Tolias/Berens/Sandberg Labs
Human MTG SMART-seq	Hodge et al.,^46^	Allen Institute for Brain Science Cell Types Database
Mouse Whole Cortex and Hippocampus SMART-seq	Yao et al.,^47^	Allen Institute for Brain Science Cell Types Database

**Software and algorithms**		

Seurat v3 package	Butler et al.,^48^ Stuart et al.,^49^	Satija Lab Seurat
Intrinsic Physiology Feature Extractor (IPFX) toolbox/patchseqtool package	Lee et al.^30^	Allen Institute for Brain Science Tools
custom code	This study	https://github.com/keon-arbabi/patch-seq-microglia

### Resource availability

#### Lead contact

Further information and requests for resources and reagents should be directed to and will be fulfilled by the lead contact, Shreejoy J. Tripathy (shreejoy.tripathy@camh.ca).

#### Materials availability

This study did not generate new unique reagents.

### Experimental model and study participant details

All datasets were obtained from the Allen Institute for Brain Science Cell Types Database (http://celltypes.brain-map.org/) or the Tolias/Berens/Sandberg labs (https://github.com/berenslab/mini-atlas).

We made use of data from three human and mouse Patch-seq experiments (Table 1).

For the human Patch-seq data from Allen Institute for Brain Science,^29^ surgical tissues were obtained from local Seattle-area hospitals and transported in NMDG-based artificial cerebral spinal fluid (ACSF) to a laboratory, where acute slices were prepared for patch clamp recording. Electrophysiological recordings were performed in warm (32–34°C) recording ACSF in the presence of pharmacological blockers of fast glutamatergic and GABAergic synaptic transmission. Upon completion of the electrophysiology experiment, negative pressure was applied to the patch pipette to extract the cytosol and nucleus of the target cell (Figure 1B). The SMART-Seq v4 platform was used for cDNA amplification and library construction, followed by sequence alignment. Single-cell transcriptomes from Patch-seq samples were subsequently mapped to a reference taxonomy based on single-nucleus RNA-seq data from the human medial temporal gyrus.^29^^,^^46^ RNA-sequencing metadata and counts data were downloaded for our analyses (accessed 05/10/2021). A total of 278 supragranular glutamatergic neurons from MTG in N = 48 subjects (37.1 ± 14.3 years, 18 female, 16 Caucasian, 1 American Indian and Alaska Native, 31 Unknown or unreported) were included. Brain slices not used for slice electrophysiology were also characterized using immunohistochemistry for various protein markers, including IBA1, marking microglia, and GFAP, marking astrocytes.

We also made use of mouse Patch-seq data from Allen Institute for Brain Science as detailed previously.^32^ Briefly, animals were anesthetized using isoflurane and intracardially perfused with ice-cold NMDG-based ACSF prior to slice preparation. Cell recordings were performed using male and female mice between the ages of P45 and P70. Multiple Cre-driver lines were used to target cells for morphoelectric and transcriptomic characterization. Electrophysiological recordings were performed in warm (34°C) recording ACSF in the presence of pharmacological blockers of fast glutamatergic and GABAergic synaptic transmission. The SMART-Seq v4 platform was used for cDNA amplification and library construction, followed by sequence alignment. Single-cell transcriptomes from Patch-seq samples were then mapped to a reference taxonomy of dissociated neuronal cells from mouse VISp.^50^ RNA-sequencing metadata and counts data were downloaded for further analyses (v2, released 07/01/2020). A total of 4,270 GABAergic interneurons sampled from visual cortex in 985 mice (445 female, P54.2 ± 6.4, C57BL/6J background strain) were included. Please see Gouwens et al. 2020 for full mouse Cre line information.

We also included mouse Patch-seq data from Scala et al.^31^ Animals were anesthetized using isoflurane and decapitated prior to rapid brain removal and collection into cold NMDG-based ACSF for slice preparation. Cell recordings were performed using male and female mice between the ages of P35 and P245. Multiple Cre-driver lines were used to target cells for morphoelectric and transcriptomic characterization. Electrophysiological recordings were performed at room temperature (25°C) using recording ACSF where fast synaptic transmission was not blocked. The Smart-seq2 protocol was used for cDNA amplification and library construction, followed by sequence alignment. Single-cell transcriptomes from patch-seq samples were mapped to multiple taxonomies, including a taxonomy based on cells from mouse VISp and anterolateral motor cortex (ALM), enabling direct comparison with cells from Gouwens et al. RNA-sequencing metadata and counts data were downloaded for further analyses (accessed 08/01/2022). A total of 1329 glutamatergic and GABAergic neurons sampled from the motor cortex in 266 mice (135 female, P84.9 ± 31.9, C57Bl/6 background strain) were included. Please see Scala et al. 2020 for full mouse Cre line infromation.

Gene expression patterns from Patch-seq samples were compared to cell dissociation-based single-cell and single-nucleus RNA-seq datasets. Hodge et al. used snRNA-seq and multiple clustering approaches to describe the cell type taxonomy of 15,928 nuclei from human MTG.^46^ Yao et al. used scRNA-seq to profile ∼1.3 million cells from the adult mouse isocortex and hippocampal formation to generate a transcriptomic cell-type taxonomy.^47^ Cells and nuclei transcriptomes quantified with SMART-seq were downloaded from both datasets and used for the present analyses (accessed 09/25/2021).

### Method details

#### Microglial contamination score

To quantify the extent of microglial contamination for each Patch-seq cell, we applied previously described methods for assessing the quality of Patch-seq transcriptomic data.^24^^,^^30^ Specifically, we employed cell type-specific markers for human and mouse neocortical neurons calculated in Lee et al. (https://github.com/AllenInstitute/patchseqtools).^30^ These markers describe both “on” markers, or genes that are highly and ubiquitously expressed in neurons of interest relative to other cell types. “Off” markers are expected to be expressed at low levels in Patch-seq cells, and if expressed together with “on” markers, are an indicator of possible cellular contamination. Lee et al. calculated both types of markers using the single-cell and single-nucleus RNA-seq datasets from dissociated cells and nuclei described previously,^46^^,^^50^ which can serve as ground truth expression data for comparison with Patch-seq cells.^30^

Using definitions described previously,^24^^,^^30^ we calculated the *contamination score* of microglia *(M)* in a neuron subtype of interest *(N)*, as:

$$CS_{N\_M}=\frac{P_{c\_N,M}-d_{N\_M}}{d_{M\_M}-d_{N\_M}}$$

The expression of microglia markers in a Patch-seq cell *c* are compared to the expected expression in reference data from which the markers were derived. To do this we take the summed normalized expression (log2 CPM) of 50 microglia markers in a cell of *N (P*_*c_N, M*_*)* and subtract the median microglia marker expression in dissociated cells of type *N (d*_*N_M*_*)*. If this numerator is negative (for example, if cell *c* expresses no microglial markers but *d*_*N_M*_ is positive), it is set to 0 in these cases (indicating no detected contamination). The denominator scales this value by the expected expression of microglial markers in reference microglia. The contamination score can thus be interpreted as a ratio of the excess microglial expression, scaled between 0 and 1 (where 1 indicates that the cell expresses microglial expression at a level similar to dissociated microglia).

#### Visualizing patch-seq gene expression

Gene expression from human and mouse Patch-seq cells was processed according to the standard Seurat V3 workflow (https://satijalab.org/seurat).^48^^,^^49^ All available cells for human and mouse were included in the following steps. Best practices for processing Patch-seq data typically involve filtering out genes that are highly expressed in non-neuronal cell types, however this step was omitted to gauge the extent of contamination. Only mitochondrial genes and genes of uncertain function were filtered out prior to normalization. The top 2000 variable features (5000 for mouse) were identified, followed by scaling, linear dimensionality reduction, and clustering using the default parameters. Cells were visualized by projection onto Uniform Manifold Approximation and Projection (UMAP) space.

#### Associations between microglial contamination and donor, cell type, and tissue characteristics

Univariate relationships between microglial contamination score and available Patch-seq metadata were described, including neuron transcriptomic-type, cell soma depth from the pial surface (in micrometers), and, in human samples, histological markers of tissue pathology. Histological pathology scores for IBA1 and GFAP protein expression were included as the only markers not skewed heavily toward zero, and were binned by marker score (low ≤1, high >1). Given the large number of mouse transcriptomic types at the cluster resolution, we aggregated closely related cell clusters by summarizing t-types using the subclass designation and the first marker gene (e.g., Sst Hspe Sema3c → Sst Hspe).

In the Gouwens and Berg datasets, we further made use of a measure of electrophysiological recording quality, quantified as the electrophysiological seal resistance in Gigaohms. In addition, we derived a relative measure of the elapsed time between subsequently recorded cells from the same donor or animal, termed “break-in time”, by defining the first cell recorded from a donor/animal as t = 0 and calculating the elapsed time in hours for each subsequent cell relative to this first cell. Both of these measures were obtained by utilizing quality control features provided within each electrophysiology data file (see section below on electrophysiology analyses).

To further explore the influence of these characteristics on microglial contamination in Patch-seq samples, we employed mixed effects models and estimated the proportion of variation in microglial contamination predicted by each variable. Only cells with data available for the variables of interest were included, and cell types with fewer than 20 cells in either the Berg or Gouwens datasets (or 10 cells in the Scala dataset) were excluded. The following model was used for the human Patch-seq data:

microglial contamination score ∼ biological sex + medical condition + scale(age) + scale(break in time) + scale(seal resistance) + scale(cell soma depth) + transcriptomic type + IBA1 protein + GFAP protein + (1 | donor id)

And for the Gouwens mouse Patch-seq data:

microglial contamination score ∼ biological sex + scale(age) + scale(break in time) + scale(seal resistance) + transcriptomic type + (1 | donor id)

And for the Scala mouse Patch-seq data:

microglial contamination score ∼ biological sex + scale(age) + scale(cell soma depth) + transcriptomic type + (1 | donor id)

#### Differential gene expression analysis

To characterize the transcriptional signature associated with microglial contamination in Patch-seq data, we performed differential expression testing. For the human data, high and low contamination groups were defined by the top and bottom quartiles of Patch-seq sampled neurons ranked by contamination score, respectively. For the mouse data, deciles were used to adjust for the smaller fraction of cells with high contamination. In each dataset, differential expression tests between these groups were performed with DESeq2^51^ using the FindMarkers function with default settings in Seurat v3. Differentially expressed genes (DEGs) were defined as having >2.5 or < -2.5 log2 fold-change and p value <0.01. In effect, this approach identifies neuronal transcripts that are significantly co-expressed with microglial contamination.

For comparison, we also performed differential expression testing between groups of microglia and neurons in the reference single-cell and single-nucleus RNA-seq datasets. We selected neuronal transcriptomic types that most closely matched those surveyed in the human and mouse Patch-seq datasets. For the Yao dataset, cells labeled “Micro-PVM” at the subclass level in all brain regions (178 cells) and “GABAergic” at the class level in VIS and VISp (6846 cells) were compared with DESeq2 as described above. For the Hodge dataset, cells labeled “Microglia'' at the subclass level (246 cells) and cells with cluster labels including “Exc L2-3”, “Exc L2-4”, and “Exc L3-4” in MTG (3609 cells) were compared. DEGs from these tests were selected as described above.

The functional context of DEGs was explored with gene set enrichment analysis. We include 19 unique microglial gene lists from 4 publications pulled from the literature, selected for their inclusion of homeostatic and activated microglia populations from both human and mouse. Friedman et al. performed a meta-analysis of purified mouse CNS myeloid cells across a wide range of disease models, and identified distinct modules of co-regulated genes associated with lipopolysaccharide (LPS) injection, interferon signaling, and neurodegeneration.^35^ Keren-Shaul et al. identified disease-associated microglia (DAM) in an AD-transgenic mouse model that potentially restricts neurodegeneration.^37^ Mathys et al. confirmed the presence of DAM and identified several other disease stage-specific microglial states in a mouse model of AD-like neurodegeneration.^38^ Olah et al. performed scRNA-seq to explore the population structure of live microglia purified from surgically resected human cortex and characterized nine clusters of microglia encompassing states specific to homeostasis, proliferation, response to cellular stress, and responses to injury and disease.^36^ These findings reflect the heterogeneity of microglia, and the presence of activation and/or disease-related subtypes. Gene sets were directly acquired from supplemental materials. If human or mouse gene symbols were not available, the provided set was converted using the getLDS() function from the biomaRt package.^52^^,^^53^ Cluster-defining genes provided by OIah et al. were filtered to those significantly downregulated in >6 other clusters to enhance specificity.

The enrichment of these gene sets in the patch-seq microglial signatures and general microglial signatures identified through differential expression testing (see results for Figure 3) was determined with the hypergeometric test implemented by the HypeR package.^54^

#### Electrophysiology analyses

Raw electrophysiological traces from human^29^ and mouse^32^ Patch-seq experiments were obtained from DANDI (dataset IDs: 000209 and 000020). Stimulation protocols consisted of long-square hyperpolarizing and depolarizing current injections. The Intrinsic Physiology Feature Extractor (IPFX) toolbox^30^ was used to extract electrophysiological features from each recorded neuron, similar to as described previously.^39^ Sweeps explicitly tagged as failed were discarded prior to feature extraction. Extracted features include subthreshold features (i.e., input resistance, sag ratio), action potential properties (i.e., action potential half-width, threshold time and voltage) derived from the rheobase spike, as well as multi-action potential spike train features derived from the IPFX-defined “hero” sweep (i.e., adaptation index). Action potential amplitude was defined as the difference between peak and threshold voltage and after hyperpolarization amplitude was defined as the difference between action potential threshold and the fast action potential trough. For electrophysiological data from the Scala mouse dataset, we employed processed electrophysiological features that were provided by the original study authors, and standardized these so that they were broadly consistent with those from the other datasets.

We used a statistical approach to ask how microglial contamination is associated with cell-to-cell variability in electrophysiological features, after controlling for other aspects of cellular and donor identity.

Specifically, Specifically, we used a mixed effects model, where, for human cells from the Berg dataset and glutamatergic and GABAergic cells from the Scala dataset:

electrophysiology feature ∼ microglial contamination score + scale(cell soma depth from pia) + (1| neuron transcriptomic type) + (1 | donor id)

And for mouse cells from the Gouwens dataset:

electrophysiology feature ∼ microglial contamination score + (1| neuron transcriptomic type) + (1 | donor id)

We used a log10 normalization to scale input resistance, rheobase, and action potential half-width and all electrophysiology features were standardized prior to modeling. We chose not to standardize microglial contamination scores to enable direct comparisons of effects between humans and mice, as there were very different standard deviations between species. Significances for the beta coefficients associated with microglial contamination were provided using the lmerTest function.^55^

### Quantification and statistical analysis

All statistical analyses were performed in the R programming language. Differential expression was performed in Seurat based on the non-parametric Wilcoxon rank sum test, and significance was defined as >2.5 or < -2.5 log2 fold-change and p value <0.01. Gene set enrichments were determined using the hypergeometric test implemented in the HypeR package,^54^ and False Discovery Rates (FDRs) were reported.

For mixed models, the coefficient of determination (R^2^) for each fixed effect (every term except donor id) was iteratively calculated by taking the difference between the marginal R^2^ value of the full model and the marginal R^2^ value of a reduced model, in which the fixed effect of interest was replaced by a random intercept. Marginal R^2^ was calculated using the method described by Nakagawa et al.^56^ and implemented in the *MuMIn* package.^57^ The variance explained by the random effect of donor id was estimated by subtracting the marginal R^2^ from the conditional R^2^ of the full model. Beta coefficients associated with microglial contamination were provided using the lmerTest function,^55^ and 95% confidence intervals and p values were calculated.

## Data Availability

• This paper analyzes existing, publicly available data. The accession numbers for the datasets are listed in the key resources table. • All original code has been deposited at Github at https://github.com/keon-arbabi/patch-seq-microglia and is publicly available as of the date of publication. • Any additional information required to reanalyze the data reported in this paper is available from the lead contact upon request.

## Supplemental information

Document S1. Figures S1–S7, Tables S3, and S4

Table S1. Human Patch-seq differential expression between cells with high vs. low microglial contamination, related to Figure 3

Table S2. Mouse Patch-seq differential expression between cells with high vs. low microglial contamination, related to Supplementary Figure 6
