# Supplementary material for: Investigating microglia-neuron crosstalk by characterizing microglial contamination in human and mouse patch-seq datasets
Source: iScience. 2023 Jul 11;26(8):107329. doi: 10.1016/j.isci.2023.107329 (PMC10374462; doi:10.1016/j.isci.2023.107329)
Supplement: Document S1. Figures S1–S7, Tables S3, and S4 [file mmc1.pdf]

iScience, Volume *26*

Supplemental information

Investigating microglia-neuron crosstalk

by characterizing microglial contamination

in human and mouse patch-seq datasets

Keon Arbabi, Yiyue Jiang, Derek Howard, Anukrati Nigam, Wataru Inoue, Guillermo Gonzalez-Burgos, Daniel Felsky, and Shreejoy J. Tripathy

Supplementary

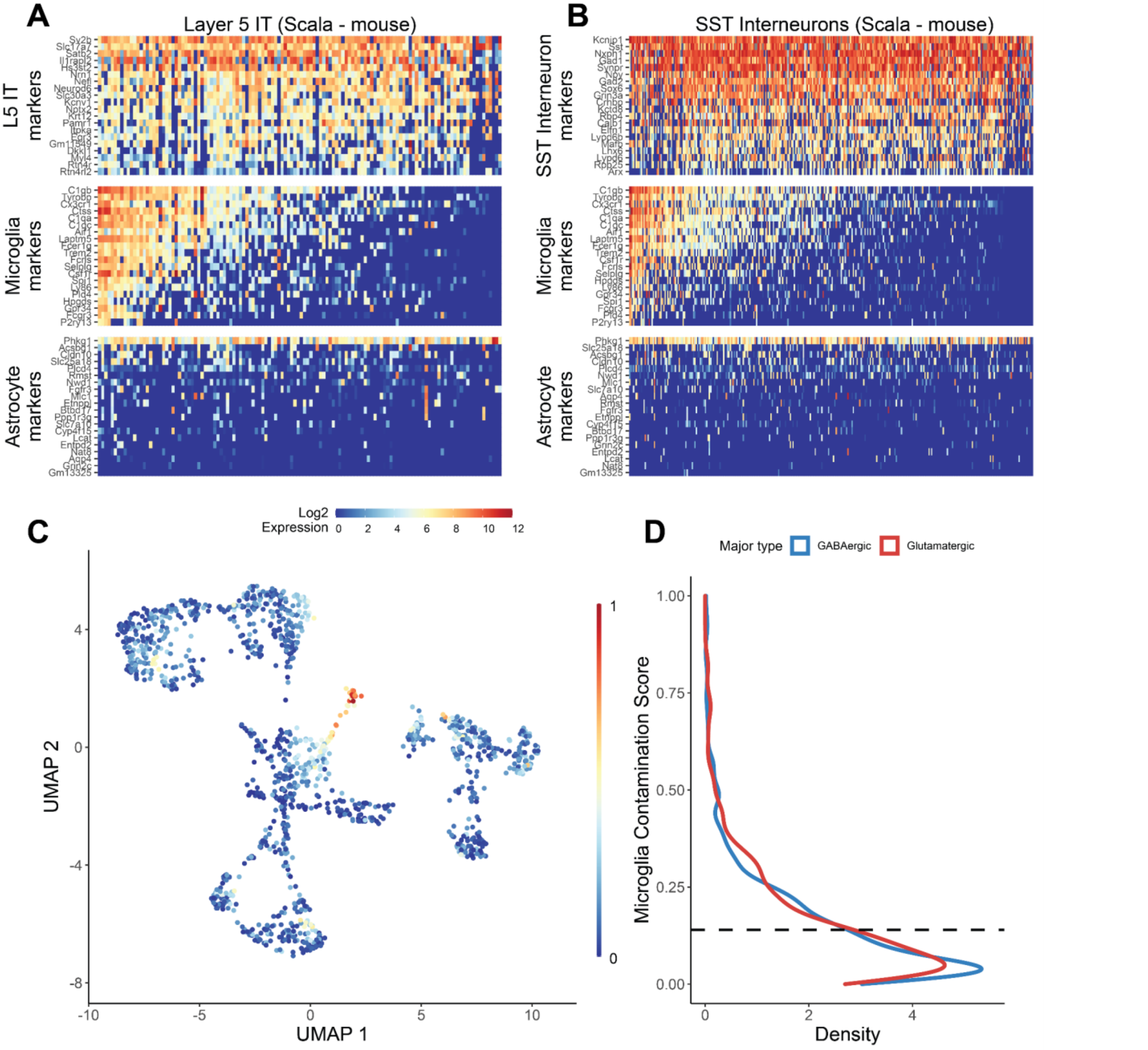

Supplementary Figure 1. ***Patch-seq transcriptomes of glutamatergic and GABAergic mouse neurons from the Scala dataset express microglial marker genes, Related to Figure 1.*** **(A,B)** Gene expression profiles for Layer 5 Intratelencephalic neurons (A) or SST interneurons (B) for various cell type-specific markers. Each row represents a cell type-specific marker gene and columns represent individual neurons, ordered from left to right by decreasing microglial contamination score. **(C)** Low-dimensional visualization of transcriptomes from neuronal Patch-seq samples clustered by most variable gene expression and color-coded by microglial contamination score. **(D)** Distribution density of microglial contamination scores for GABAergic (blue) or glutamatergic (red) neurons. Dashed lines indicate population mean.

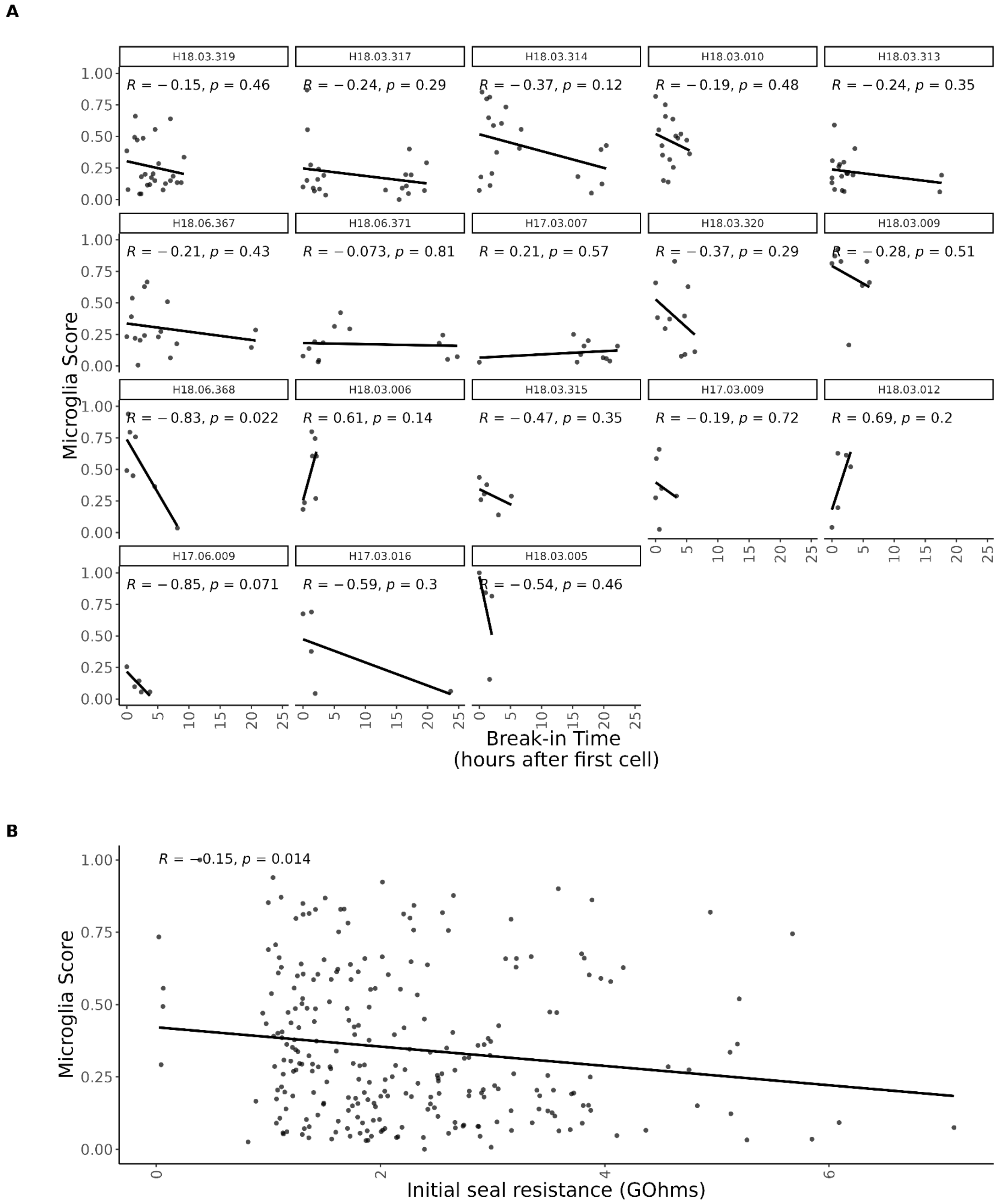

Supplementary Figure 2. ***Association between microglial contamination and relative break-in time for each cell per donor and recording seal resistance for recorded cells from the Berg human dataset, Related to Figure 2.*** A) The time in hours relative to the time at which the first cell per donor was patched (x-axis) compared to microglial contamination score (y-axis). B) Initial seal resistance of each recorded cell (x-axis).

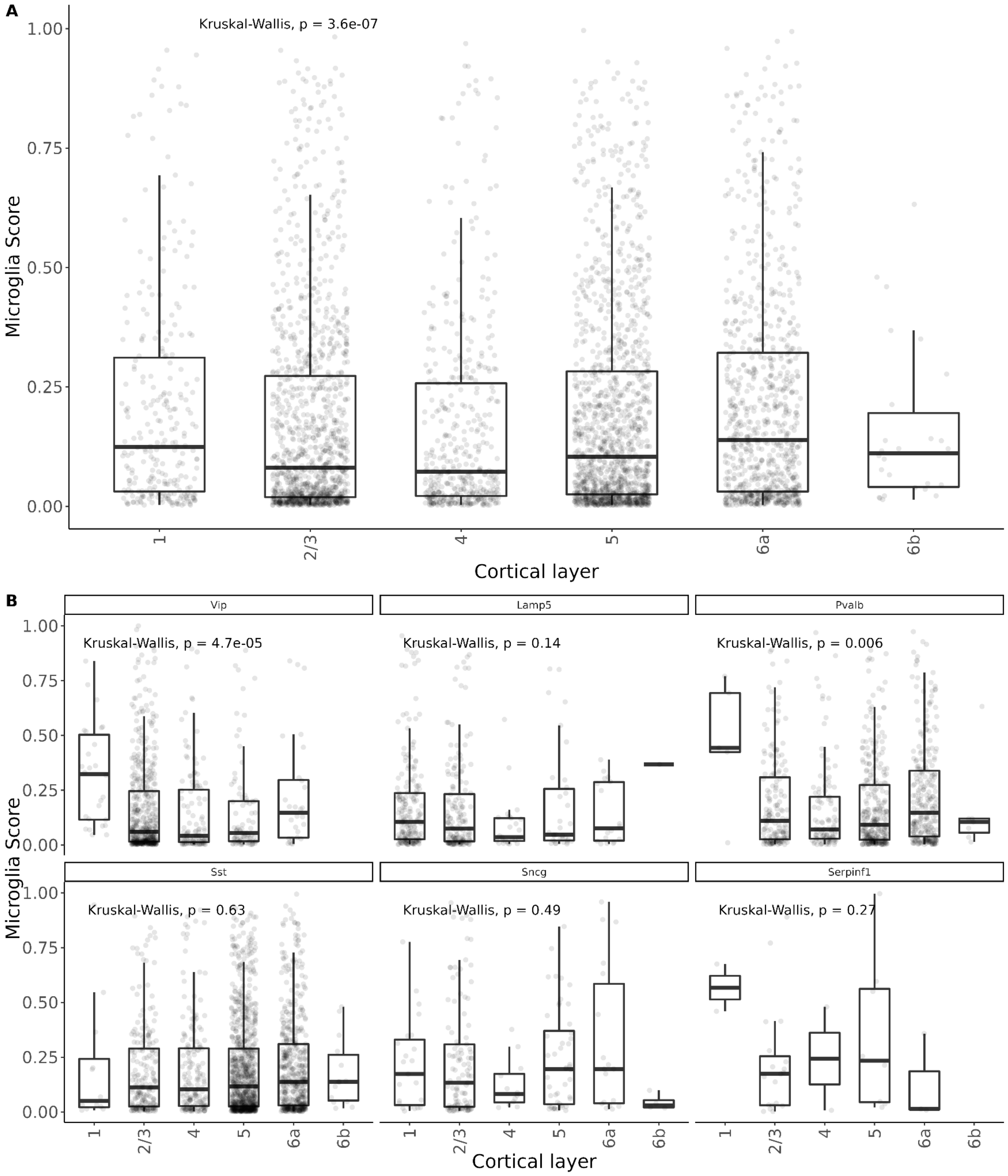

Supplementary Figure 3. ***Association between microglial contamination and neocortical layer for mouse GABAergic cells from the Gouwens dataset, Related to Figure 2*** A) Microglial contamination scores (y-axis) versus neocortical layer (x-axis) among all GABAergic cells sampled in the Gouwens dataset. B) Same as A, but faceted by subclass.

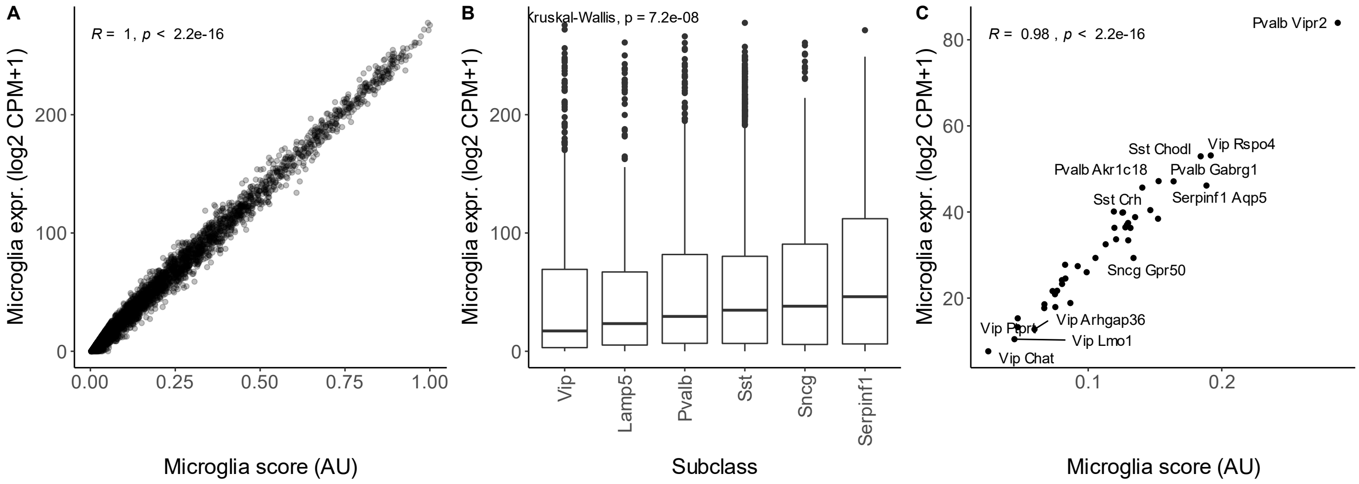

Supplementary Figure 4. ***Summed expression of microglia-specific markers (cell type-independent) is highly correlated with microglial contamination score (cell type-dependent) and exhibits differences across Patch-seq sampled GABAergic cell types from mouse, Related to Figure 2*** **(A)** Correlation between summed normalized expression (log2 CPM+1) of microglia-specific marker genes for each Patch-seq sampled cell from the Gouwens mouse dataset (y-axis) and microglial contamination score (x-axis) ($R = 1$, $p < 2.2 * 10^{-16}$). The difference between these measures is that microglial contamination score is scaled by the amount of expected microglial transcripts for each respective cell type in dissociated cells. Summed expression of microglia-specific markers allows for comparisons between cell types by avoiding the cell type-dependence of microglial contamination scores. **B)** Boxplots depicting the association between summed expression of microglia-specific markers (y-axis) in mouse GABAergic Patch-seq cells and interneuron cell type identity summarized at the cluster level (x-axis) (Kruskal-Wallis, $p = 7.2 * 10^{-8}$). **C)** Correlation between summed expression of microglia-specific markers (y-axis) and microglial contamination score (x-axis) summarized at the subclass level ($R = 0.98$, $p < 2.2 * 10^{-16}$).

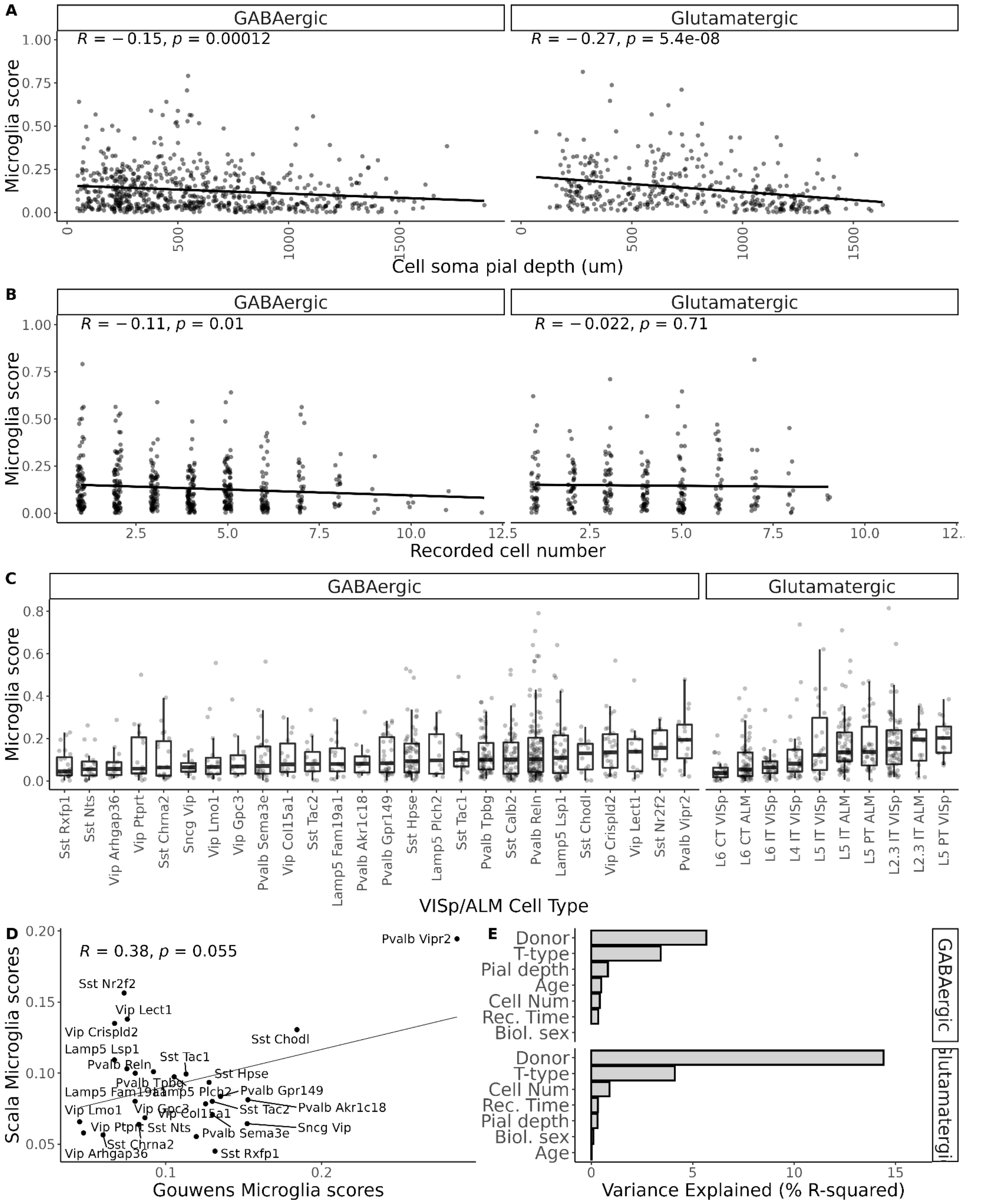

Supplementary Figure 5. ***Associations between cell soma pial depth, cell type, and other factors with microglial expression in Patch-seq samples from the Scala mouse dataset, Related to Figure 2.*** **(A)** Associations between microglial contamination scores (y-axis) estimated from mouse GABAergic and Glutamatergic neuron Patch-seq samples with recorded depths of cell soma from the pial surface (x-axis). Inset

correlations reflect Pearson's correlations and line indicates best linear fit. **(B)** Associations between microglial contamination scores (y-axis) and cell type identity summarized at the t-type or cluster level (mapped to the same VISp/ALM cell type atlas used in the Gouwens dataset). **(C)** Comparison of cell type-specific Microglia scores among GABAergic cells from the Scala dataset (y-axis) and Gouwens dataset (x-axis). Each dot reflects median microglial contamination scores for one transcriptomically-defined cell type. Inset correlation reflects Pearson's correlation and line indicates best linear fit. **(D)** Estimated percent variance explained (R-squared) in microglial contamination scores among GABAergic (top) and Glutamatergic (bottom) samples by various factors, including donor/animal identity, neuronal cell type identity (t-type), age, sex, and sample depth from pial surface (Pial depth).

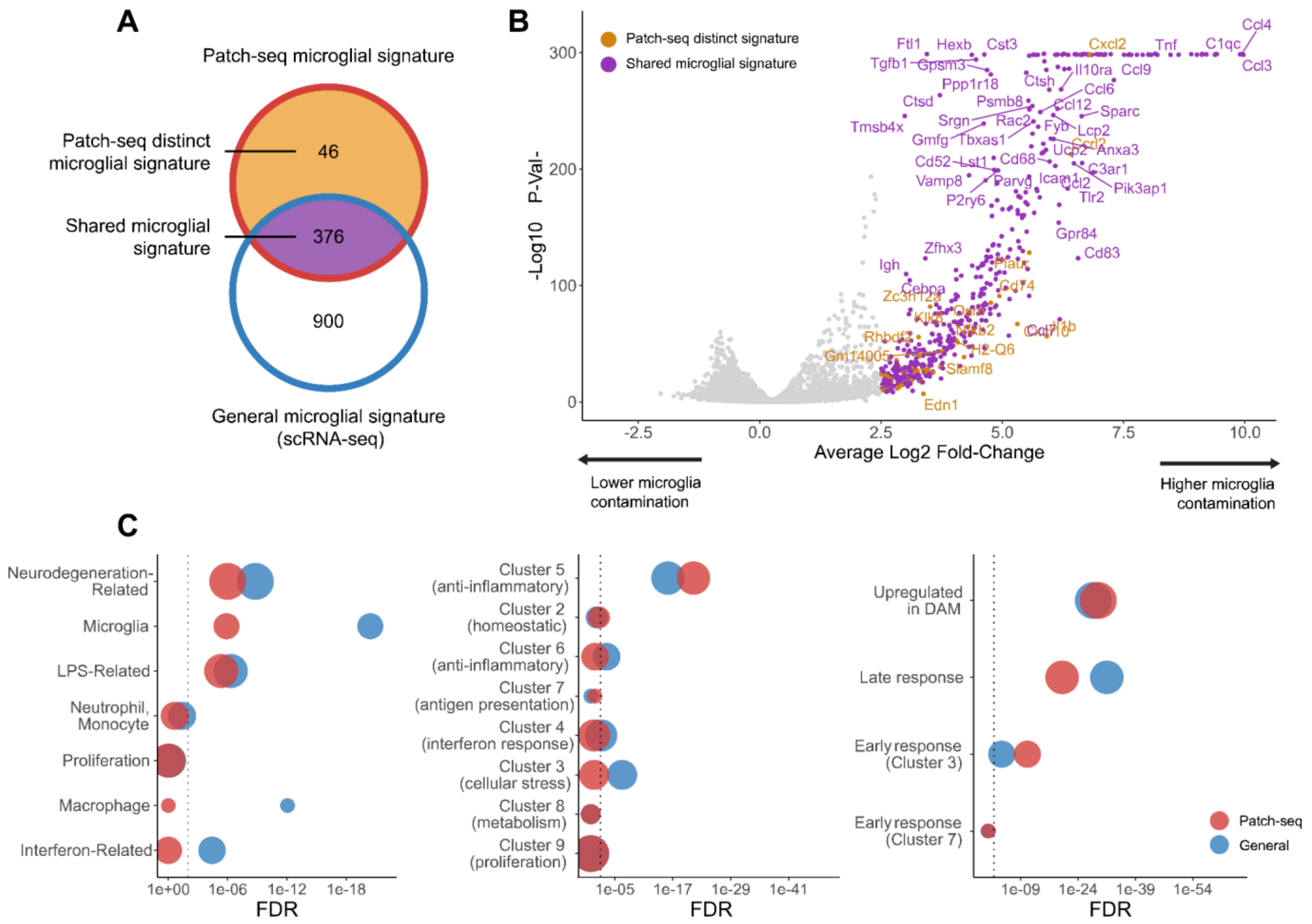

Supplementary Figure 6. ***Microglial contamination in mouse Patch-seq reflects a distinct transcriptional signature related to microglia activation, Related to Figure 3.*** **(A)** Venn diagram indicating the number of genes defining various transcriptional signatures: the signature of general microglia in mouse dissociated single-cell data (blue border), the signature of Patch-seq microglial contamination in the Gouwens mouse neuronal Patch-seq dataset (red border), genes that are shared between the Patch-seq microglial and general microglia signatures (purple fill), and genes that are distinct to the Patch-seq microglial signature that are not also present in the transcriptional signatures of general microglia (yellow fill). **(B)** Volcano plot of transcriptional signature of Patch-seq microglial contamination, illustrating differentially expressed genes in Patch-seq datasets between mouse neuronal samples with high vs. low microglial contamination. Points denote differentially expressed genes (log2 fold-change >2.5; p-value < 0.01), and colors are as in (A). Genes with -log10 P Values at 300 indicate significance levels beyond the machine precision limit. **C)** Enrichment analysis of general microglia (blue) and Patch-seq microglia (red) transcriptional signatures (as in A) intersected with gene sets of diverse microglial phenotypes and states from multiple data sources. Dot size reflects the number of genes in each gene set. Dotted line is FDR = 0.05.

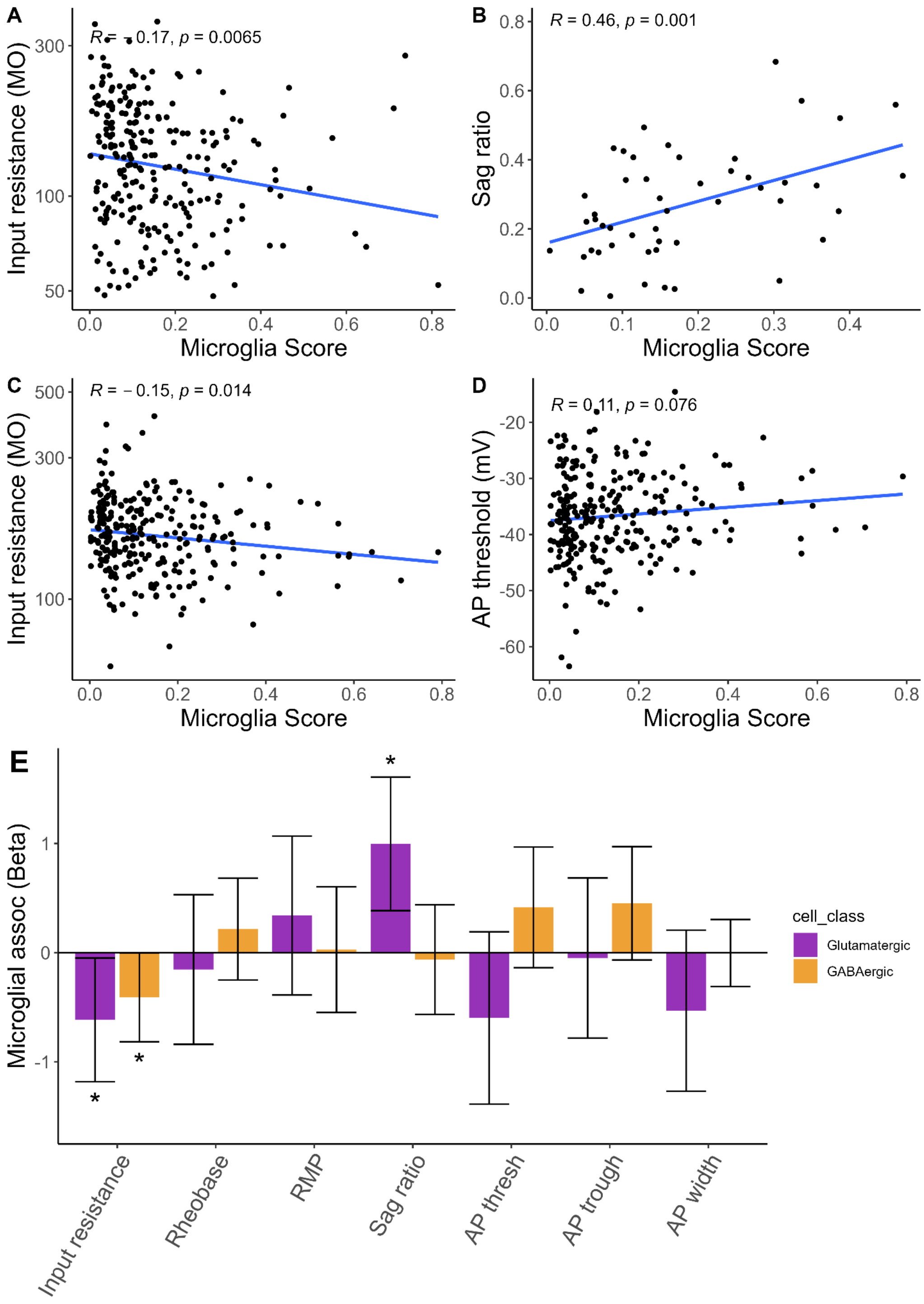

Supplementary Figure 7*. Associations between microglial contamination and neuronal intrinsic electrophysiology in the Scala mouse dataset, Related to Figure 4*. (A-D) Scatter plots illustrating electrophysiological features (y-axis) versus microglial contamination scores (x-axis). Lines reflect lines of best fit and inset correlation values denote Pearson's correlations. (A) Input resistance values from intra-telencephalic pyramidal cells; (B) Sag ratio values from extra-telencephalic pyramidal cells; (C, D) Input resistance values (C) and action potential threshold values (D) from Pvalb interneurons. **(E)** Association between microglial contamination and electrophysiological characteristics, as estimated using a mixed effects model. Bars indicate

effect sizes (Beta coefficients) and error bars denote 95% confidence intervals. Asterisks denote beta coefficients where $p < 0.05$ (ANOVA). Negative (positive) beta coefficients indicate increased microglial contamination is associated with a decrease (increase) in the electrophysiological property. Electrophysiological features have been standardized to unit variance, enabling comparison of beta coefficient effect sizes between species.

Supplementary Table 3. ***Effect sizes for associations between microglial contamination and electrophysiological features from mixed effects models in AIBS datasets, Related to Figure 4***

Ephys property	***Species***	***Estimate***	***Std error***	***Statistic***	***DF***	***P-value***
Input resistance	*human*	*-0.6877*	*0.1933*	*-3.5578*	*245.3263*	*0.0004*
Input resistance	*mouse*	*-0.3150*	*0.0464*	*-6.7953*	*4121.6742*	*0.0000*
Rheobase	*human*	*0.5690*	*0.1804*	*3.1549*	*249.0130*	*0.0018*
Rheobase	*mouse*	*0.1501*	*0.0443*	*3.3875*	*4135.1570*	*0.0007*
AP width	*human*	*-0.1708*	*0.2069*	*-0.8256*	*249.3981*	*0.4098*
AP width	*mouse*	*0.0080*	*0.0401*	*0.2001*	*4084.8713*	*0.8414*
AP thresh	*human*	*0.5924*	*0.2609*	*2.2709*	*246.4383*	*0.0240*
AP thresh	*mouse*	*0.2118*	*0.0660*	*3.2097*	*4120.5277*	*0.0013*
AP trough	*human*	*0.6546*	*0.2499*	*2.6190*	*250.8830*	*0.0094*
AP trough	*mouse*	*0.2943*	*0.0603*	*4.8773*	*4129.7096*	*0.0000*
FI slope	*human*	*-0.1940*	*0.2222*	*-0.8729*	*249.1397*	*0.3835*
FI slope	*mouse*	*-0.1032*	*0.0548*	*-1.8845*	*4137.7518*	*0.0596*
RMP	*human*	*0.1581*	*0.2251*	*0.7023*	*250.0758*	*0.4831*
RMP	*mouse*	*0.2390*	*0.0668*	*3.5785*	*4147.1071*	*0.0003*
Sag ratio	*human*	*0.3735*	*0.2123*	*1.7596*	*236.7804*	*0.0798*
Sag ratio	*mouse*	*-0.0712*	*0.0582*	*-1.2233*	*4136.6600*	*0.2213*

s

Supplementary Table 4. ***Effect sizes for associations between microglial contamination and electro-physiological features from mixed effects models in Scala dataset, Related to Supplementary Figure 7***

Ephys property	***Species***	***Estimate***	***Std error***	***Statistic***	***DF***	***P-value***
Input resistance	***Glutamatergic***	*-0.6155*	*0.2890*	*-2.1299*	*374.6893*	*0.0338*
Input resistance	***GABAergic***	*-0.4090*	*0.2077*	*-1.9688*	*643.9142*	*0.0494*
Rheobase	***Glutamatergic***	*-0.1541*	*0.3494*	*-0.4410*	*376.2763*	*0.6595*
Rheobase	***GABAergic***	*0.2158*	*0.2380*	*0.9068*	*635.6736*	*0.3648*
AP width	***Glutamatergic***	*-0.5312*	*0.3763*	*-1.4116*	*374.1334*	*0.1589*
AP width	***GABAergic***	*-0.0028*	*0.1565*	*-0.0181*	*637.7115*	*0.9856*
AP thresh	***Glutamatergic***	*-0.5981*	*0.4024*	*-1.4863*	*383.3698*	*0.1380*
AP thresh	***GABAergic***	*0.4151*	*0.2821*	*1.4713*	*627.8247*	*0.1417*
AP trough	***Glutamatergic***	*-0.0490*	*0.3742*	*-0.1310*	*368.4694*	*0.8959*
AP trough	***GABAergic***	*0.4522*	*0.2648*	*1.7077*	*634.0137*	*0.0882*
RMP	***Glutamatergic***	*0.3403*	*0.3711*	*0.9170*	*372.5816*	*0.3598*
RMP	***GABAergic***	*0.0281*	*0.2937*	*0.0956*	*648.0092*	*0.9239*
Sag ratio	***Glutamatergic***	*0.9961*	*0.3121*	*3.1915*	*369.8386*	*0.0015*
Sag ratio	***GABAergic***	*-0.0634*	*0.2564*	*-0.2471*	*642.0564*	*0.8049*
